# Sensitivity Enhancement of Strain Sensing Utilizing a Differential Pair of Fiber Bragg Gratings

**DOI:** 10.3390/s120403891

**Published:** 2012-03-26

**Authors:** Zhiyong Zhang, Lianshan Yan, Wei Pan, Bin Luo, Ping Wang, Likang Guo, Wei Zhou

**Affiliations:** 1 Center for Information Photonics & Communication, School of Information Science and Technology, Southwest Jiaotong University, Chengdu, Sichuan 614202, China; E-Mails: zhiyongzhang@home.swjtu.edu.cn (Z.Z.); wpan@home.swjtu.edu.cn (W.P.); bluo@home.swjtu.edu.cn (B.L.); zhouwei20060808@126.com (W.Z.); 2 Lab of Railway Engineering, School of Civil Engineering, Southwest Jiaotong University, Chengdu, Sichuan 614202, China; E-Mails: wping@home.swjtu.edu.cn (P.W.); guolk@home.swjtu.edu.cn (L.G.)

**Keywords:** fiber Bragg grating, strain measurement, differential pair of FBG, sensitivity, railway safety monitoring

## Abstract

In strain measurement applications, the matched fiber Bragg gratings (FBG) method is generally used to reduce temperature dependence effects. The FBG parameters have to be designed to meet the requirements by the particular application. The bandwidth and slope of the FBG has to be balanced well, according to the measurement range, accuracy and sensitivity. A sensitivity enhanced strain demodulation method without sacrificing the measurement range for FBG sensing systems is proposed and demonstrated utilizing a pair of reference FBGs. One of the reference FBGs and the sensing FBG have almost the same Bragg wavelength, while the other reference FBGs has a Bragg wavelength offset relative to the sensing FBG. Reflected optical signals from the sensing FBG pass through two reference FBGs, and subtract from each other after the detection. Doubled strain measurement sensitivity is obtained by static rail load experiments compared to the general matched grating approach, and further verified in dynamic load experiments. Experimental results indicate that such a method could be used for real-time rail strain monitoring applications.

## 1. Introduction

Optical fiber sensors (OFSs) have advantages of small dimensions, high sensitivity, and immunity against electromagnetic interference (EMI) [1–3]. Therefore, OFSs have been widely used for structural health monitoring applications in transportation, civil structures, aerospace, marine and smart structures, *etc.* [3–14].

Driven by the high-speed transportation network, the demand for railway safety monitoring is of great importance for various situations. Although many techniques based on electronics or manual operation are still being used, OFS is proven to be more efficient and attractive, especially in harsh environments. A variety of OFS have been developed for strain and temperature measurements, including the distributed techniques based on the Brillouin, Raman or Rayleigh scattering methods and the discrete fiber sensors based on FBGs [1].

The simplest detection scheme for the FBG sensor is the reflection matched grating method, which uses the reflection and transmission power spectra of two FBGs [1,15]. This method has the advantages of fast measurement and low cost, but it is mainly used for the applications where precision is not of concern, such as axes counting in railways [1] or the tunnel transport system. For more accurate strain detection using FBG sensors, the tunable laser source or Fabry-Perot filter is often used with its drawback of relatively high cost.

In railway applications, real-time rail strain monitoring at certain key locations is very important. For example, partial loading of a high-speed train may cause a huge disaster. For such applications, both the measurement sensitivity and dynamic range are key parameters [16,17], although typically one has to balance the sensitivity (*i.e.*, narrower bandwidth of FBG) and the dynamic range (*i.e.*, wider bandwidth of FBG).

In this paper, a demodulated method based on a differential pair of FBGs (DP-FBG) is proposed and demonstrated. The reference FBGs are used in a pair: one of the reference FBGs with the same Bragg wavelength as the sensing FBG, while the other reference FBGs has a Bragg wavelength offset relative to the sensing FBG. Reflected optical signals from the sensing FBG pass through the two reference FBGs individually and are detected through simple power measurements. Using such scheme, small strain values or variations can be detected by subtracting the two signals from the reference gratings. The DP-FBG demodulation hardware and software are further verified utilizing static and dynamic rail load experiments.

## 2. Configurations and Principle

According to Bragg's law, the reflected center wavelength (or Bragg wavelength) *λ_B_* of a FBG sensor is given by [1,15–19]:

(1) $$\lambda_{B}=2n_{\text{eff}}\Lambda$$

where Λ is the Bragg grating period and *n_eff_* is the effective refractive index of the fiber core. Both Λ and *n_eff_* may vary (*i.e.*, ΔΛ and Δ*n_eff_*) due to dynamic strain or temperature.

The general configuration of the matched grating for strain measurement by [1,15] utilizes two FBGs. One of the FBGs is used as the sensing unit mounted on the object, while the other one is used as the reference unit without external stress. The dynamic wavelength shift of the sensing FBG is demodulated by a matched filter function after simple power detection [1,15]. The major advantage of such configuration is its independence of the environmental temperature.

**Figure 1.**
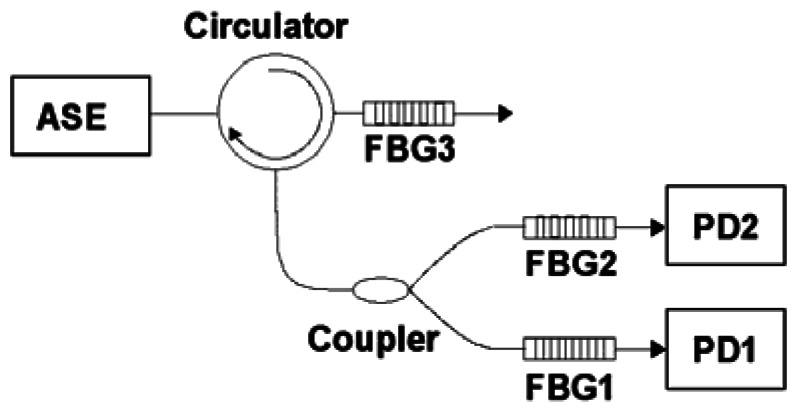
Scheme of the DP-FBG demodulation method.

The configuration of a differential pair of FBGs (DP-FBG) demodulation method is shown in Figure 1. The difference between the DP-FBG method and the general matched grating method is that the latter one uses an extra Bragg grating that is not matched with the sensing FBG.

An ASE broadband light source injects the optical power into the fiber through a circulator. Then the back-reflected light from the sensing unit (FBG3) is divided into two parts by a coupler. One arm passes through the matched Bragg grating FBG1 (the Bragg wavelength of it is the same as that of FBG3) and the other passes through the mismatched FBG2 (the Bragg wavelength of it has an offset against that of FBG3). The optical signals are then detected by two photo-detectors (PD1 and PD2). Finally the strain demodulation (measurement) is accomplished by subtracting the signal detected by PD1 from the signal detected by PD2, or *vice versa*.

**Figure 2.**
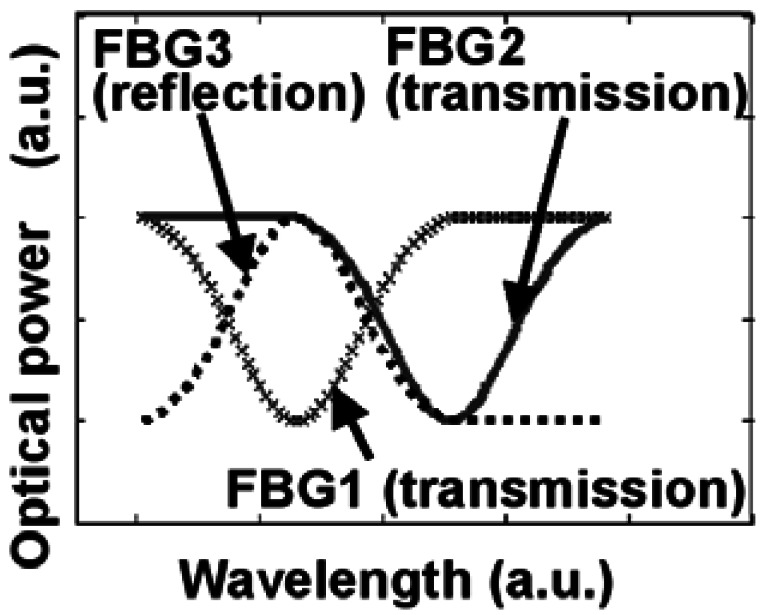
Optical spectra of three FBGs (FBG1&FBG2: reference FBGs; FBG3: sensing FBG).

The reflection spectrum function of a FBG can be modeled as a Gaussian function with a Bragg wavelength [15]. Assuming the center wavelength of FBG1 (*λ*_1_) is equal to that of FBG3 (*λ*_3_), which is shown in Figure 2. But the Bragg wavelength of FBG2 (*λ*_2_) is located at the minimum value of the reflection spectrum of FBG3. The reflection optical spectrum distribution [15] of FBG3 is given by:

(2) $$I_{s}(\lambda )=S\left(\lambda_{B}\right)G_{3}(\lambda )$$

where *S*(*λ_B_*) is the spectrum characteristic of the ASE light source, which should has a smoothly spectrum profile, and *G*_3_(*λ*) is the Gaussian model of the reflection function of FBG3. The total power reflected by FBG3 is the integral of Equation (2) over all the wavelength range.

After passing through the circulator, the reflected lights transmit through FBG1 and FBG2. Hence the received optical power by PD1 and PD2 are described as:

(3) $$\{\begin{array}{c} P_{PD1}=k_{1}\int_{0}^{+\infty }S\left(\lambda_{B}\right)G_{3}\left(\lambda \right)T_{1}\left(\lambda \right)d\lambda \\ P_{PD2}=k_{2}\int_{0}^{+\infty }S\left(\lambda_{B}\right)G_{3}\left(\lambda \right)T_{2}\left(\lambda \right)d\lambda \end{array}$$

Where *k*_1_ and *k*_2_ are scale factor, *T*_1_(*λ*) *=* 1 − *G*_1_(*λ*) is the transmission function of FBG1, and *T*_2_(*λ*) *=* 1 − *G*_2_(*λ*) is the transmission function of FBG2.

**Figure 3.**
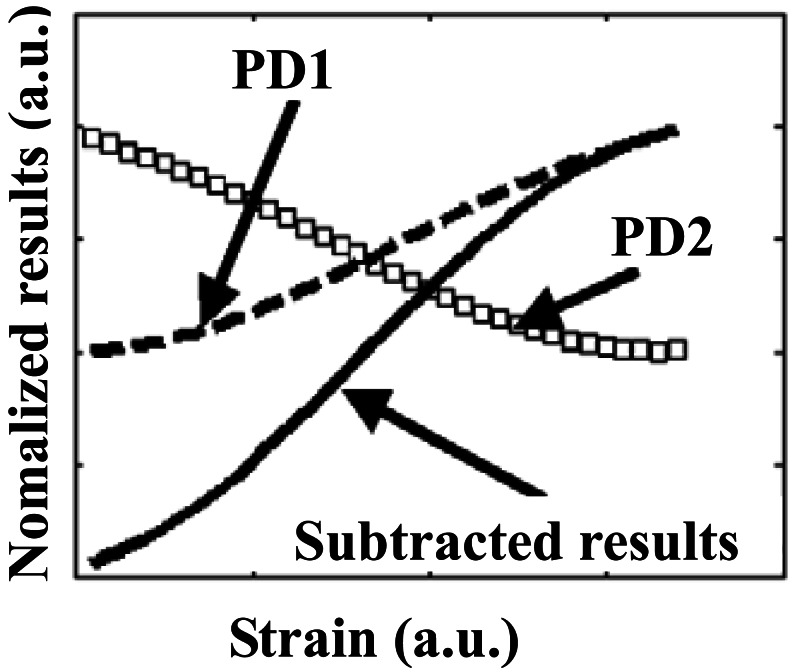
Principle of DP-FBG demodulation with detected power subtraction.

The relationship between the detected optical power and the corresponding sensed strain is shown in Figure 3. The dashed curve and the circle-marked curve indicate the optical power detected by PD1 and PD2, respectively, while the solid curve represents the results of the DP-FBG demodulation, *i.e.*, subtracted values from PD1 and PD2. It is obvious that the slope of the demodulation curve obtained by the DP-FBG scheme is almost double of that from either PD1 or PD2, indicating that the strain sensitivity of the new scheme is twice of the general matched grating method in ideal situation.

In addition to the enhanced sensitivity using the scheme of two PDs detection, there is one more advantage. By knowing the optical power from two detected signals, we could reduce or even diminish the effect of power fluctuations of the light source (ASE) through a simple normalization procedure.

## 3. Experimental Results and Discussion

**Figure 4.**
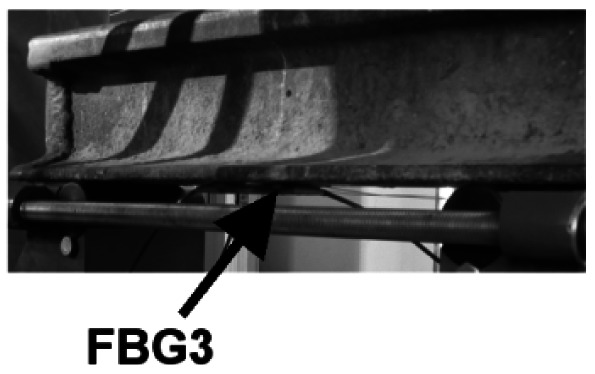
Mounted FBG on the rail.

The DP-FBG demodulation method is experimentally verified by rail strain measurements. Following the scheme shown in Figure 1 with signal acquisition and processing units, we built a setup as shown in Figure 4. The sensing FBG3 is bonded to the bottom of the rail, and two reference FBGs are placed near the rail without external stress. Note that the temperature difference between the rail setup and the lab environment during our experiments is negligible, however, for practical railway applications, there might be up to tens of degrees difference, especially during the hot summer time. Therefore, it would be necessary to make sure that all three FBGs experience the same temperature. To do that, we can either: (i) mount the reference FBGs close to the sensing FBG utilizing the temperature-sensitive packaging method (*i.e.*, one end of the FBG is mounted while the other one is released), or (ii) use the same package as the sensing FBG but with the direction perpendicular to the sensing FBG (*i.e.*, reference FBGs won't experience the stress variations).

**Figure 5.**
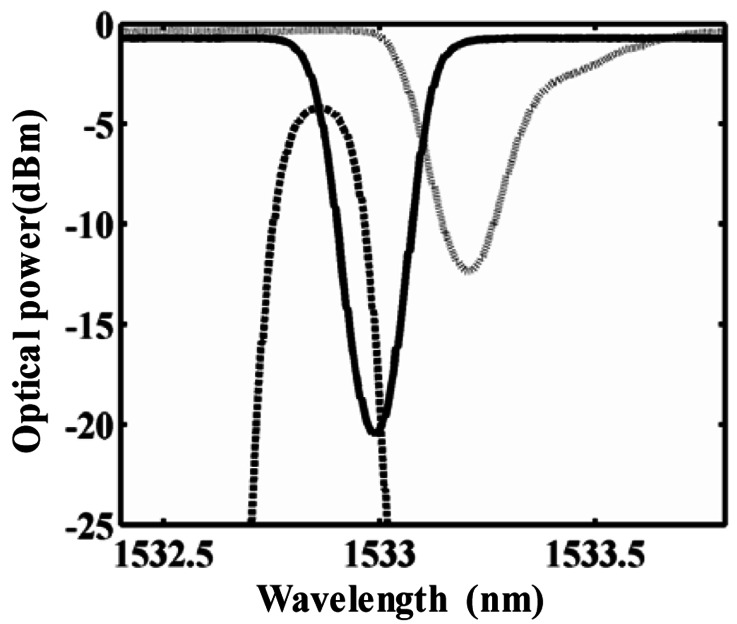
Measured optical spectra of three FBGs (Solid-line: FBG1; dotted-line: FBG2; dashed-line: FBG3).

The normalized optical spectra of three FBGs are given in Figure 5 (as measured by an Aritsu optical spectrum analyzer). The solid line indicates the transmission spectrum of FBG1, the dotted line represents the transmission spectrum of FBG2, and the dashed line corresponds to the reflection spectrum of FBG3. Three FBGs have almost the same 3-dB bandwidth of ∼0.24 nm. The center wavelength of reference FBG1 and FBG2 are 1,532.97 nm and 1,533.26 nm, respectively, with a wavelength difference of about 0.28 nm.

**Figure 6.**
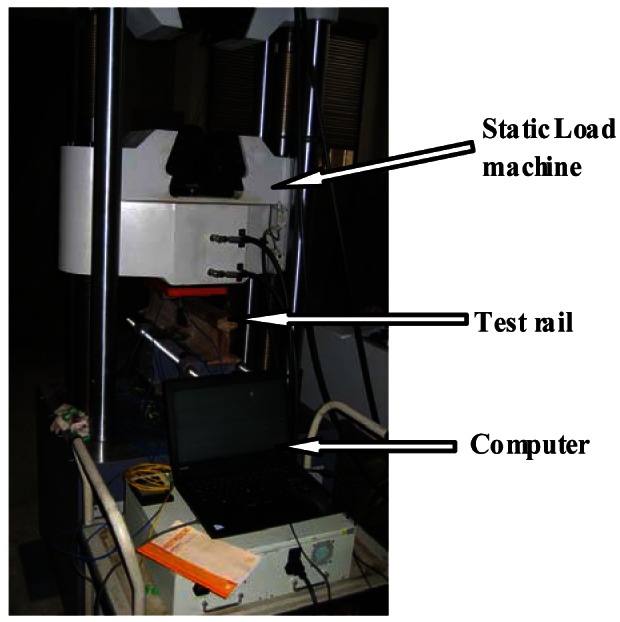
Static rail load platform.

The Bragg wavelength of FBG3 is 1,532.85 nm, a negative offset about 0.12 nm from that of FBG1. The center wavelength shift of FBG3 may be caused by the residual stress after it is bonded to the rail. The programmable load is added on the rail by a static load machine, as shown in Figure 6. Note that, when the center wavelength of FBG 3 is located out of the span between the center wavelengths FBG1 and FBG2, the output optical power through the branch of FBG1 will first decrease and then increase with the load added and the measurement range will not be started at zero kN.

**Figure 7.**
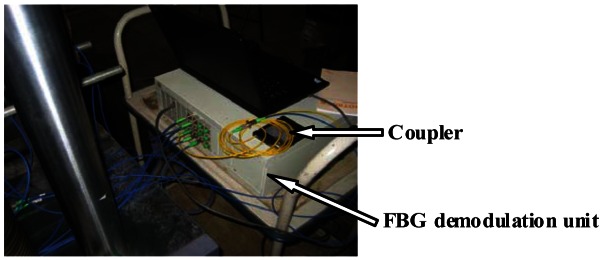
FBG demodulation system.

The optical signal's acquisition and data preprocessing device are developed using the LP1768 ARM chip with an embedded 12 bit A/D unit. These data are transferred to a PC through the TCP/IP protocol with a data read out speed ∼1 kHz, and then converted into the real voltage signals. Finally, these voltage values can be converted to the corresponding dynamic strain values after calibration. The whole demodulation system is shown in Figure 7.

**Figure 8.**
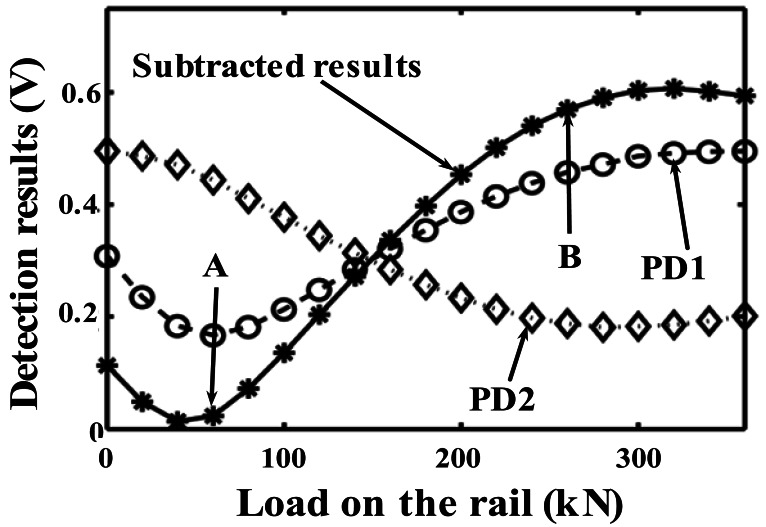
Detected signal values of PD1 and PD2 as a function of the rail load.

**Figure 9.**
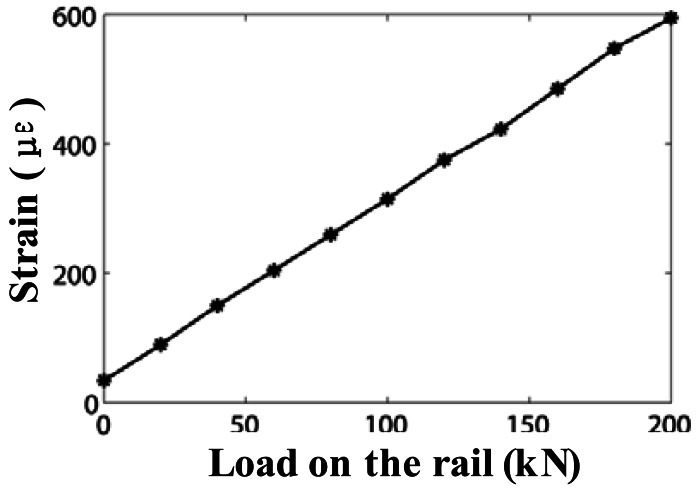
Static results by the strain gauge with load from 0 to 200 kN.

The normalized experimental results are shown in Figure 8. The circle symbols with dashed line are the normalized optical powers detected by PD1, and the diamond shapes with dotted line are the results obtained from PD2, therefore the star symbols with solid line represent results of the DP-FBG method. Note that, there is a DC offset voltage about 0.17 V in the measurement results of both PD1 and PD2. And a DC offset voltage about 0.3 V is added on the subtracted results. Figure 9 illustrates the measurement results by a strain gauge system with the load range from 0 to 200-kN.

The slope of the measurement results from 60-kN to 260-kN, using the DP-FBG method in Figure 8, is nearly twice as large as that using the general matched FBG method. Therefore, the measurement sensitivity is increased substantially using the DP-FBG demodulation scheme. Measurement results by the DP-FBG method from 60-kN to 260-kN (from point A to point B in Figure 8) exhibit good linearity, therefore it can be calibrated using results of the strain gauge in Figure 9. When the load is more than 300-kN, the demodulation result exhibits certain ambiguity, which indicates the limit (measurement range) of such approach. Note that there is a 60-kN induced strain caused by the mismatch of the center wavelength of the sensing FBG (FBG3), and it can be diminished after calibration. For most rail applications, a 200-kN induced strain (after the preload strain being calibrated) on one wheel-rail is sufficient large (this corresponds to the weight of one carriage with eight wheels being about 200 kN × 8 = 1,600 kN). If the Bragg wavelength of the sensing FBG could be well selected or adjusted after being mounted, the linear demodulation range and the measurement range could be further optimized.

**Figure 10.**
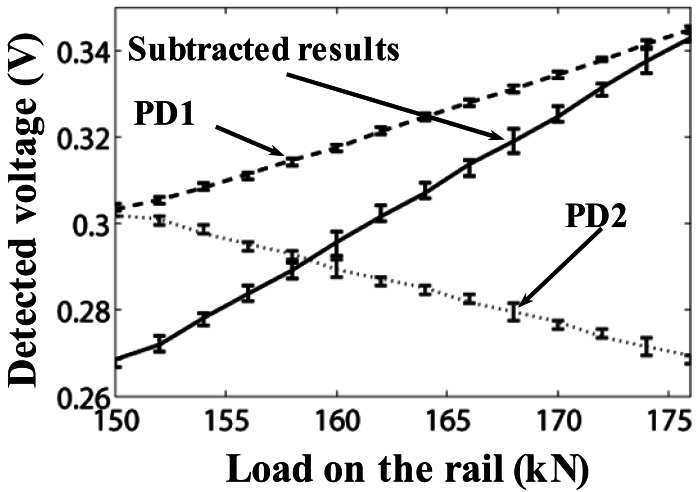
Static results with load from 150 to 176 kN.

Figure 10 shows the measured voltages of PDs (*i.e.*, PD1 and PD2) as a function of the applied load on the rail from 150 to 176 kN with a step of 2 kN. Each point on the curves is obtained from three hundred samples with slight variations. Mean, maximum and minimum values of these samples are illustrated. Results with dashed line are the detected voltage by PD1, results with dotted gray line are obtained by PD2, and results with solid line are the subtracted ones. Data deviations may be caused by unrelated electrical noise from detection circuits.

From Figure 10, we can clearly see that the measurement sensitivity (*i.e.*, the slope of demodulation results) is significantly enhanced (typically twice that of the conventional matched grating approach) without sacrificing the dynamic range (generally limited by the small value of either FBGs' bandwidth).

**Figure 11.**
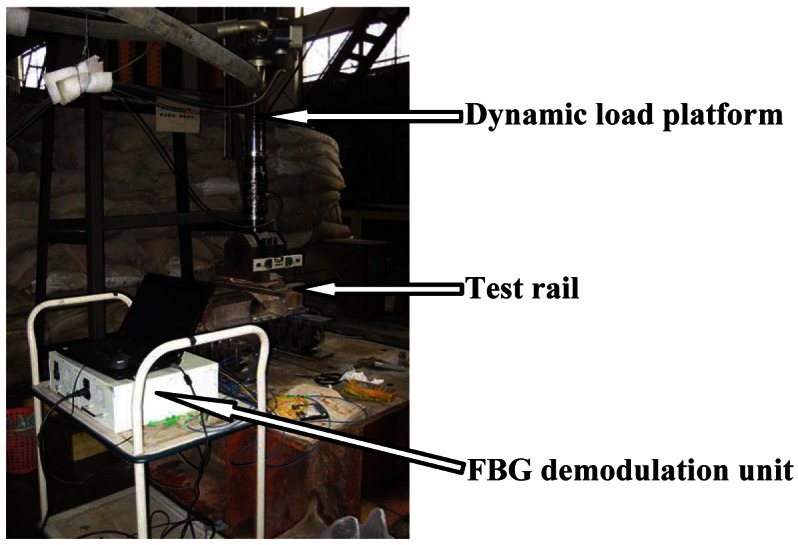
Dynamic rail load platform.

To evaluate the dynamic performance of this scheme, we apply periodic strain on the rail with a frequency of 3 Hz or 6 Hz. The range of load is from 40 to 220 kN. The dynamic load platform is shown in Figure 11, with the test rail placed on a steel table.

**Figure 12.**
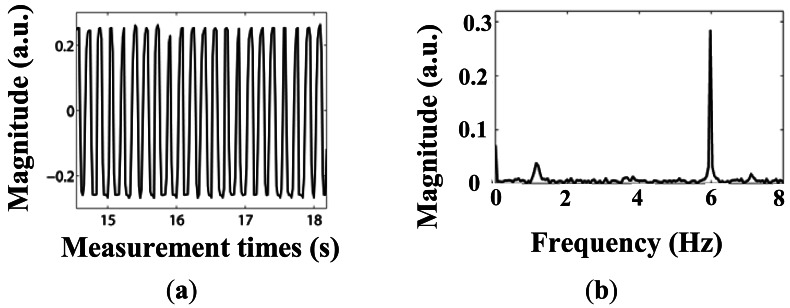
(**a**) Dynamic load experiment results under vibration frequency of 3 Hz; (**b**) Signal frequency analysis under vibration frequency of 3 Hz.

**Figure 13.**
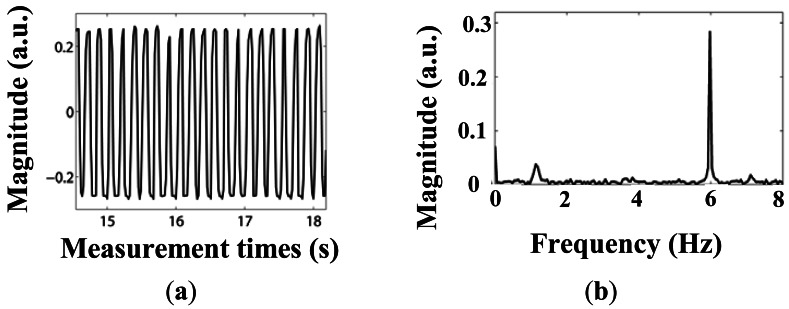
(**a**) Dynamic load experiment results under vibration frequency of 6 Hz; (**b**) Signal frequency analysis under vibration frequency of 6 Hz.

Results of the dynamic loading are illustrated in Figures 12 and 13. The demodulation unit can work well when the vibration frequency is either 3 Hz or 6 Hz.

The existing noise frequency peak ∼1.2 Hz in Figure 13 is generated by the vibration of the platform itself (similar results were found in our previous measurements using the strain gauge, not shown here).

## 4. Conclusions

A DP-FBG demodulation method is proposed and demonstrated. Without sacrificing the dynamic range, the measurement sensitivity of strain can be improved by a factor of almost 2 compared to the conventional matched FBG demodulation scheme. Both static and dynamic load experiments illustrate the effectiveness of the proposed DP-FBG approach. Such demonstrations indicate that the system can be potentially used for real time rail monitoring, such as the axle counting, load unbalance detection of two rails, and vibration signatures monitoring at selected locations in railway applications.
